# Superconductivity in a 122-type Fe-based compound (La,Na,K)Fe_**2**_**As**_**2**_

**DOI:** 10.1038/s41598-018-34265-2

**Published:** 2018-11-14

**Authors:** Kenji Kawashima, Shigeyuki Ishida, Hiroshi Fujihisa, Yoshito Gotoh, Yoshiyuki Yoshida, Hiroshi Eisaki, Hiraku Ogino, Akira Iyo

**Affiliations:** 1 IMRA Material R&D Co., Ltd., 2-1 Asahi-machi, Kariya, Aichi 448-0032 Japan; 2 National Institute of Advanced Industrial Science and Technology (AIST), 1-1-1 Umezono, Tsukuba, Ibaraki 305-8568 Japan

## Abstract

We synthesized a Fe-based superconductor (FeSC), (La,Na,K)Fe_2_As_2_, and characterized its superconducting properties. It was found that (La,Na,K)Fe_2_As_2_ has a 122-type (ThCr_2_Si_2_-type) structure with a space group *I*4/*mmm* (No. 139), identical to (Ba,K)Fe_2_As_2_ and (La,Na)Fe_2_As_2_ but distinct from so-called 1144-type FeSCs such as CaKFe_4_As_4_ and (La,Na)CsFe_4_As_4_. The results demonstrate that the formation of the 1144-type phase necessitates the large ionic radius mismatch among the so-called *A*-site constituent elements of the *A*Fe_2_As_2_ formula. The lattice constants are *a* = 3.850(1) Å and *c* = 13.21(1) Å. The La, Na, and K ions occupy the same atomic site of Wyckoff position 1*a*. Electrical resistivity and magnetic susceptibility show the superconducting transition at 22.5 K. The transition temperature (*T*_c_) of (La,Na,K)Fe_2_As_2_ is comparable with that of 122-type (La,Na)Fe_2_As_2_ and 1144-type (La,Na)*A*Fe_4_As_4_ (*A* = Rb, Cs), while being more than 10 K lower than those of typical 122- and 1144-type FeSCs. The results suggest that the random distribution of La^3+^ and Na^+^ ions is the main reason for lower *T*_c_ in the *AE* = (La,Na) 122-type and 1144-type FeSCs.

## Introduction

The discovery of superconductivity in LaFeAs(O,F) in 2008 has triggered the search for new Fe-based superconductors^1^. A large number of Fe-based superconductors (FeSCs) with various crystal structures have been reported, such as *Ln*FeAs(O,F) (*Ln* corresponds to rare earth elements) — a 1111-type compound^1–3^, (*AE*_1−*x*_*A*_*x*_)Fe_2_As_2_ (*AE* = Ca, Sr, Ba, Eu, *A* = Na, K, Rb) — a 122-type compound^4,5^, *AEA*Fe_4_As_4_ (*AE* = Ca, Sr, Ba, Eu, *A* = Na, K, Rb) — a 1144-type compound^6–9^, *A*FeAs (*A* = Li, Na) — a 111-type compound^10^, (Ca,*Ln*)FeAs_2_ — a 112-type compound^11,12^, and perovskite Fe nictide^13^. The superconducting transition temperature (*T*_c_) reaches as high as ~55 K in bulk materials, and several reports have indicated that *T*_c_ is close to 100 K in thin film samples^14,15^.

Usually, the 122-type and the 1144-type compounds contain divalent (mostly alkali earth) elements. On the other hand, we have recently demonstrated that the combination of monovalent Na and trivalent La successfully substitute the divalent ions and form the 122- and 1144-type superconductors, such as La_0.5−*x*_Na_0.5+*x*_Fe_2_As_2_ ((La,Na)Fe_2_As_2_) and (La,Na)*A*Fe_4_As_4_ (*A* = Rb, Cs). For 122-type (La,Na)Fe_2_As_2_, La_0.4_Na_0.6_Fe_2_As_2_ (*x* = 0.1) is a non-superconductor and exhibits a structural phase transition at 130 K^16^. Superconductivity appears between $0.15\,\leqq \,x\,\leqq \,0.35$ as a consequence of hole carrier doping, with the highest *T*_c_ of 27.0 K for *x* = 0.3^17^. On the other hand, 1144-type (La,Na)*A*Fe_4_As_4_ exhibit superconductivity by themselves, with *T*_c_ around 25 K for (La,Na)RbFe_4_As_4_ and 24 K for (La,Na)CsFe_4_As_4_, respectively^18^.

The previous results indicate that the combinations of (La,Na) yields the 122-type structure while those of (La,Na,Rb) and (La,Na,Cs) yield the 1144-type structure. From the results, we have pointed out two parameters which possibly determine the preferred crystal structure. The first parameter is the ionic size difference between the *AE* ions and the *A* ions, namely, *Δr* = *r*_AE_ − *r*_A_, where *r*_AE_ and *r*_A_ are ionic radii of *AE* (=Ca, Sr, Ba, Eu, (La,Na)) and *A* (=Na, K, Rb, Cs) ions. Here large *Δr* favors the 1144-type structure. The second parameter is the lattice mismatch of the original 122-type compound crystal structure, namely, *Δa* = |*a*_AE122_ − *a*_A122_|, where *a*_AE122_ and *a*_A122_ are the lattice constants of *AE*Fe_2_As_2_ and *A*Fe_2_As_2_. Here small *Δa* favors the 1144-type structure. According to the criteria, (La,Na) prefers the 122-type while (La,Na,Rb(Cs)) prefers the 1144-type, respectively.

The combination of (La,Na,K) is interesting in the above regard, since its parameters are close to the border of the above criteria. More specifically, the 122-type structure is expected judging from *Δr*, while the 1144-type is favored based on *Δa*. In order to extend the material verify of FeSCs, and more importantly, to determine which parameter indeed governs the crystal structure of real materials, we tried to synthesize (La,Na,K)Fe_2_As_2_. In this paper, we show that (La,Na,K)Fe_2_As_2_ forms the 122-type crystal structure. The observed *T*_c_ is 22.5 K, which is close to that of 122-type (La,Na)Fe_2_As_2_ and 1144-type (La,Na)*A*Fe_4_As_4_ (A = Rb, Cs) FeSCs. We discuss the phase stability of (La,Na,K)Fe_2_As_2_ and possible reasons for their lower *T*_c_ based on the experimental observations.

## Results

**Figure 1:**
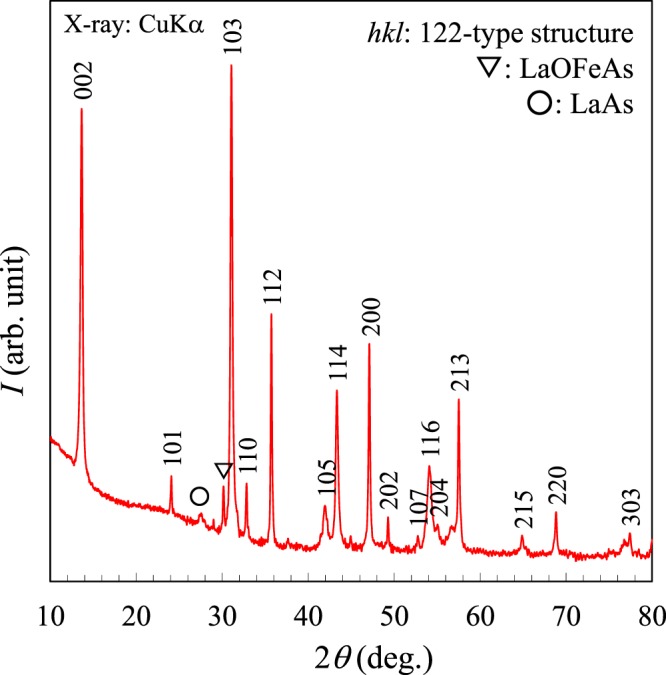
Powder X-ray patterns of (La,Na,K)Fe_2_As_2_.

Figure 1 shows the powder X-ray diffraction (PXRD) pattern of the synthesized sample. The main phase can be indexed as a tetragonal unit cell with a space group *I*4/*mmm* (No. 139), which corresponds to the 122-type (ThCr_2_Si_2_-type) structure. Extra reflections, assigned to a minor impurity phase, are identified as LaAs and LaOFeAs (non-superconducting phase)^1,3,19^. Most importantly, there are no peaks corresponding to *h* + *k* + *l* = odd number, which are the signature of the 1144-type compounds. La, Na, and K occupy the same atomic position (Wyckoff position 1*a*) in its crystal structure. The lattice constants of (La,Na,K)Fe_2_As_2_ are *a* = 3.850(1)Å and *c* = 13.21(1)Å. The *a* and *c* lattice parameters of (La,Na,K)Fe_2_As_2_ are between those of La_0.4_Na_0.6_Fe_2_As_2_ (*a* = 3.8669(1) Å and *c* = 12.108(1) Å) and KFe_2_As_2_ (*a* = 3.8414(1) Å and *c* = 13.837(1) Å)^16,17,20^. More specifically, *a*-axis lengths of these compounds are: KFe_2_As_2_ < (La,Na,K)Fe_2_As_2_ < (La,Na,K)Fe_2_As_2_, while *c*-axis lengths are (La,Na)Fe_2_As_2_ < (La,Na,K)Fe_2_As_2_ < KFe_2_As_2_, respectively.

We synthesized samples with various La/Na ratio and determined their lattice parameters and the chemical compositions (Table S1 and Fig. S1 in Supplementary Information). It turned out that the actual chemical composition does not depend on the initial compositions and yields close to La_0.2_Na_0.3_K_0.5_Fe_2_As. Correspondingly, the lattice constants do not depend on the initial composition (Table S1 and Fig. S2). Indeed, the change of the lattice constant is, if any, smaller by one order or more compared with the case of (La,Na)Fe_2_As_2_^17^. The results suggest that the 122-type (La,Na,K)Fe_2_As_2_ is stable only under fixed chemical composition. We note that *T*_c_ shows no La/Na (nominal) composition dependence, in contrast to the continuous change in *T*_c_ for 122-type (La,Na)Fe_2_As_2_^17^.

**Figure 2:**
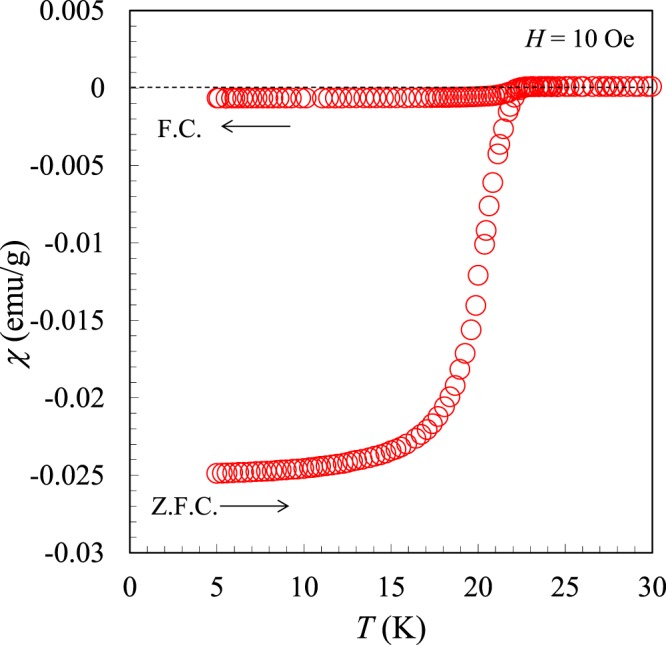
Temperature dependence of the magnetic susceptibility of (La,Na,K)Fe_2_As_2_.

Figure 2 shows the temperature (*T*) dependence of the magnetic susceptibility of (La,Na,K)Fe_2_As_2_ under an applied magnetic field of *H* = 10 Oe. The magnetic susceptibility exhibits a marked drop at 22.5 K in both ZFC and FC processes. Since the possible impurity phase LaAs and LaOFeAs do not show superconductivity^1,3,19^, the superconductivity comes from the main (La,Na,K)Fe_2_As_2_ phase. The shielding volume fraction is 102%, a reasonable value as a bulk superconductor^21^.

**Figure 3:**
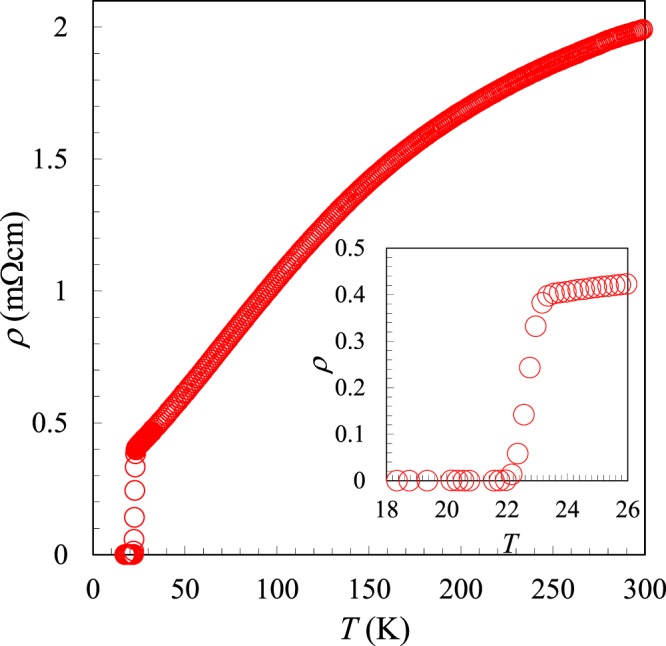
Temperature dependence of the electrical resistivity of (La,Na,K)Fe_2_As_2_. Inset shows a magnified view near *T*_c_.

Figure 3 shows the electrical resistivity of (La,Na,K)Fe_2_As_2_ as a function of *T*. The resistivity data shows the metallic behavior with convex curvature down to low-*T*. This behavior is also observed in other 122- and 1144-type superconductors^4–9,17,18^. The residual resistivity ratio, *RRR* = *ρ*_300_/*ρ*_0_, is 5.21, indicating the absence of strong scattering arising from impurities and/or grain boundaries, suitable for investigating their physical properties. As seen in the inset of Fig. 3, the resistivity sharply decreases at 23 K and reaches zero resistivity at 22 K.

**Figure 4:**
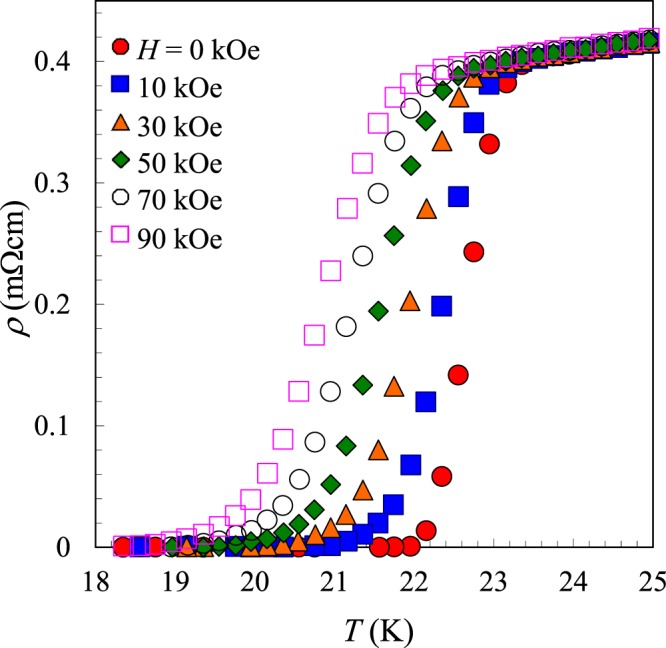
Temperature dependence of the electrical resistivity of (La,Na,K)Fe_2_As_2_ under various magnetic fields up to 90 kOe.

**Figure 5:**
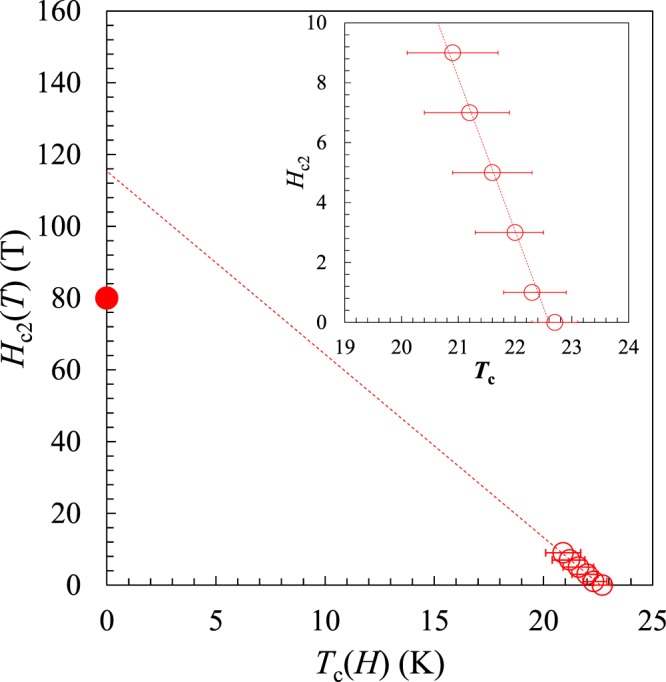
*H*-*T* phase diagram of (La,Na,K)Fe_2_As_2_. Dotted lines show the linear fitting result. Inset shows a magnified view near *T*_c_.

Figure 4 shows the electrical resistivity data for (La,Na,K)Fe_2_As_2_ under various magnetic fields (*H*) as functions of *T*. The onset *T*_c_ and the zero resistivity temperature decrease systematically with increasing *H*. The transition width does not change with *H* up to the highest *H*. The superconducting transitions are not completely suppressed under *H* ≦ 90 kOe, indicating that the upper critical field of *H*_c2_ is very large. A plot of *H*_c2_(*T*) versus *T*_c_(*H*) is shown in Fig. 5. Here *T*_c_(*H*) is defined as the midpoint of the superconducting transition for each *H*, and the horizontal bar indicates the *T*-range between 10% and 90% of the resistivity transition. *H*_c2_(*T*) shows the linear *T*-dependence within the measured *T*- and *H*- range. The slope, *dH*_c2_/*dT*, is −5.015 T/K. Using the Werthamer-Helfand-Hohenberg (WHH) formula, *H*_c2_(0) = −0.69 (*dH*_c2_/*dT*)|_*T*c_*T*_c_ for a type-II superconductor^22^, the upper critical magnetic field at 0 K is estimated to be 80 T. The corresponding coherence length (*ξ*_0_) is calculated to be 20.3 Å, estimated from the relationship between *H*_c2_ ~ Φ_0_/2π*ζ*_0_^2^, where Φ_0_ is quantum flux.

## Discussion

**Figure 6:**
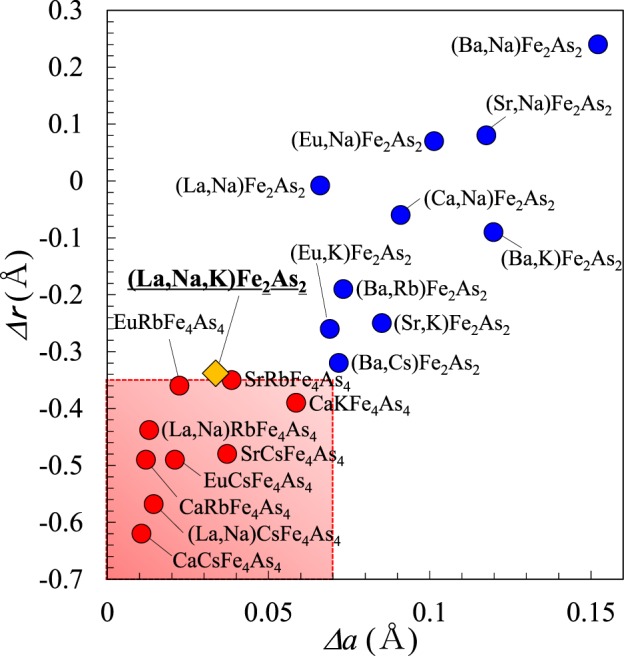
Plot of the difference between the ionic radius (VIII) of *AE*^2+^ and *A*^+^ (*Δr* = *r*_AE_ − *r*_A_) and the difference (absolute value) between the *a*-axis lengths of *AE*Fe_2_As_2_ (*AE*122) and *A*Fe_2_As_2_ (*A*122) (*Δa* = |*a*_AE122_ − *a*_A122_|) for 122- and 1144-type Fe-based superconductors^4–9,17,18^. Yellow diamond shows (La,Na,K)Fe_2_As_2_.

The present study demonstrates that (La,Na,K)Fe_2_As_2_ crystalize into a 122-type crystal structure rather than a 1144-type structure. Figure 6 shows the variation of the 122-type and 1144-type compounds in terms of the ionic size (VIII) difference of *Δr* = *r*_AE_ − *r*_A_ (*r*_AE_ and *r*_A_ are ionic radii of *AE* and *A* ions) and the lattice constant difference of the end materials, *Δa* = |*a*_AE122_ − *a*_A122_|, (*a*_AE122_ and *a*_A122_ are the *a*-axis lattice constants of *AE*Fe_2_As_2_ and *A*Fe_2_As_2_). From previous case studies, we have shown that the 1144-type structure is formed when *Δa* < 0.07 Å and *Δr* < −0.35 Å. In the case of (La,Na,K)Fe_2_As_2_, *Δa* = |*a*_(La,Na)122_ − *a*_K122_| is 0.03 Å, which possibly favors the 1144-type structure. On the other hand, *Δr* = *r*_(La,Na)_ − *r*_K_ = −0.34 Å, which is at the boundary between the 122-type and the 1144-type phases. Giving that (La,Na,K)Fe_2_As_2_ forms the 122-type structure, one can conclude that the critical parameter which determines the real crystal structure is *Δr*, rather than *Δa*.

**Figure 7:**
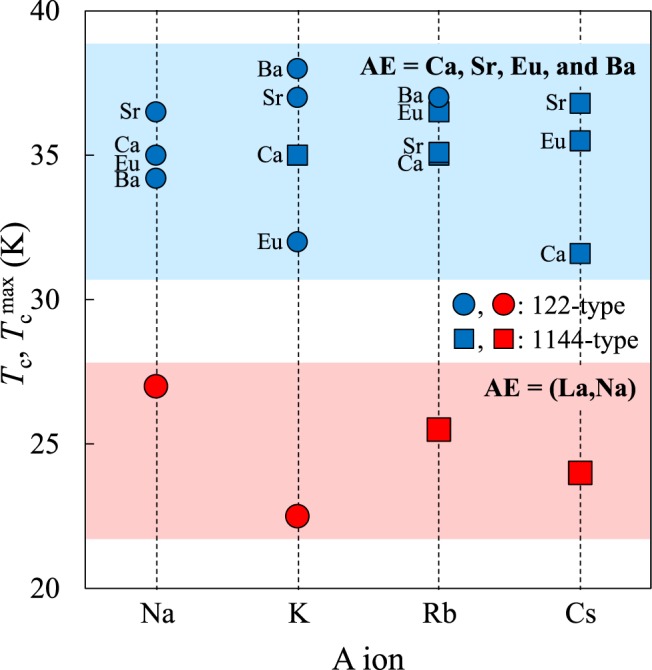
*T*_c_^max^ of 122-type *AE*_1−*x*_*A*_*x*_Fe_2_As_2_ (circles) and *T*_c_ of 1144-type *AEA*Fe_4_As_4_ (squares) (*AE* = Ca, Sr, Eu, Ba, (La,Na)) as a function of alkali metal element (*A* = Na, K, Rb, Cs).

In Fig. 7 we summarize *T*_c_^max^ of 122- and 1144-type superconductors with various alkali metal elements (*A* = Na, K, Rb, Cs). Clearly *T*_c_ with *AE* = (La,Na) is significantly lower, by nearly 10 K, than other 122- and 1144-type FeSCs. Furthermore, *T*_c_ of (La,Na,K)Fe_2_As_2_ is 22.5 K, much lower than *T*_c_ = 27 K for (La,Na)Fe_2_As_2_. In our previous paper, we pointed out two possible origins for lower *T*_c_ in the materials containing *AE* = (La,Na)^18^, namely, the difference in the As-FeAs bond angles, and the random distribution of trivalent La^3+^ and monovalent Na^+^ ions, which possibly causes the strong potential disorder and/or local lattice distortion^23^. The local lattice distortion is even larger for (La,Na,K)Fe_2_As_2_ because of the large contrast in the ionic radii of La^3+^ (*r*_La_ = 1.16 Å), Na^+^ (*r*_Na_ = 1.18 Å) and K^+^ (*r*_K_ = 1.51 Å)^24^. The present results suggest that the random distribution of the *A*/*AE* ions is responsible for the low *T*_c_ in the *AE* = (La,Na) 122-type and 1144-type FeSCs.

## Conclusion

We synthesized (La,Na,K)Fe_2_As_2_, which has a 122-type structure with *T*_c_ = 22.5 K. This case study successfully demonstrate that namely, the formation of the 1144-type crystal structure requires sufficient amount of ionic radius difference between the constituent *AE* (=Ca, Sr, Eu, (La,Na)) and *A* (=Na, K, Rb, Cs) ions. *T*_c_ of (La,Na,K)Fe_2_As_2_ is much lower than that of other 122- and 1144-type FeSCs that do not include trivalent La^3+^ in their composition, and is comparable but slightly lower compared with (La,Na)Fe_2_As_2_ and (La,Na)*A*Fe_4_As_4_ (*A* = Rb, Cs), presumably due to the mixture of three elements (La,Na,K) with different valence/ionic radius within a same site.

## Methods

### Preparation of (La,Na,K)Fe_2_As_2_ samples

Polycrystalline samples of (La,Na,K)Fe_2_As_2_ were synthesized using the stainless steel (SS) pipe and cap method^6,17,18^ as we have employed in synthesizing the 122-type and the 1144-type compounds. First, the precursors, LaAs, *A*As (*A* = Na, K), FeAs, and Fe_2_As, were prepared via the reaction of La, *A*, or Fe with As. Then the precursor powders were mixed in a molar ratio of La:Na:K:Fe:As = 0.4:0.6:1:4:4 (nominal composition: La_0.2_Na_0.3_K_0.5_Fe_2_As_2_, ((La,Na,K)Fe_2_As_2_) together with 5 at% excess *A*As (*A* = Na, K) to compensate for the evaporation of *A* and As during the heating process. The mixed powder was pressed into a pellet and put into a SS pipe, which was then sealed using tube-fitting caps. The process was carried out in a nitrogen-filled glove box. The SS pipe was heated to 1143 K for 2 h in a box furnace and quenched to room temperature.

### Material characterization

The synthesized sample was characterized by powder X-ray diffraction using a diffractometer with CuKα radiation (Rigaku, Ultima IV) equipped with a high-speed detector system (Rigaku, D/teX Ultra). Intensity data were collected with CuKα radiation over a 2*θ* range from 5° to 80° at a 0.01° step width. The compositions of the samples were analyzed by an energy dispersive X-ray spectrometry (SwiftED3000) equipped in an electron microscope (TM3000, Hitachi High-Technologies). Magnetic susceptibility measurements were performed using a SQUID magnetometer (Quantum Design, MPMS-XL) at temperatures from 5 to 50 K under an applied magnetic field of *H* = 10 Oe. This measurement was carried out on warming after zero-field cooling (ZFC process) and then on cooling in a field (FC process). The electrical resistivity was measured using a conventional DC four-probe method at temperatures from 5 to 300 K at applied magnetic fields up to 90 kOe, using PPMS (Quantum Design).

## Electronic supplementary material

Supplementary Information
