# Supplementary material for: Superconductivity in a 122-type Fe-based compound (La,Na,K)Fe2As2
Source: Sci Rep. 2018 Nov 14;8:16827. doi: 10.1038/s41598-018-34265-2 (PMC6235837; doi:10.1038/s41598-018-34265-2)
Supplement: Supplementary file 1 — Supplementary Information [file 41598_2018_34265_MOESM1_ESM.pdf]

Supplementary information

Superconductivity in a 122-type Fe-based compound $(La,Na,K)Fe_2As_2$

Kenji Kawashima[1,2*], Shigeyuki Ishida[2], Hiroshi Fujihisa[2], Yoshito Gotoh[2], Yoshiyuki Yoshida[2], Hiroshi Eisaki[2], Hiraku Ogino[2], and Akira Iyo[2]

[1]IMRA Material R&D Co., Ltd., 2-1 Asahi-machi, Kariya, Aichi 448-0032, Japan
[2]National Institute of Advanced Industrial Science and Technology (AIST), 1-1-1 Umezono, Tsukuba, Ibaraki 305-8568, Japan

Table S1. lattice constant and determined composition of $(La_{0.5-x}Na_xK_{0.5})Fe_2As_2$

x	Nominal composition	Lattice constants		Determined composition
		a (Å)	c (Å)	
0.3	$La_{0.2}Na_{0.33}K_{0.55}Fe_2As_{2.18}$	3.850(1)	13.23(1)	$La_{0.15(2)}Na_{0.28(4)}K_{0.54(4)}Fe_{2.02(3)}As_{2.01(4)}$
0.25	$La_{0.25}Na_{0.275}K_{0.55}Fe_2As_{2.18}$	3.853(1)	13.24(1)	$La_{0.21(3)}Na_{0.31(4)}K_{0.51(3)}Fe_{1.98(4)}As_{1.99(5)}$
0.2	$La_{0.3}Na_{0.22}K_{0.55}Fe_2As_{2.18}$	3.853(1)	13.23(1)	$La_{0.19(3)}Na_{0.25(2)}K_{0.53(4)}Fe_{2.00(5)}As_{2.03(3)}$

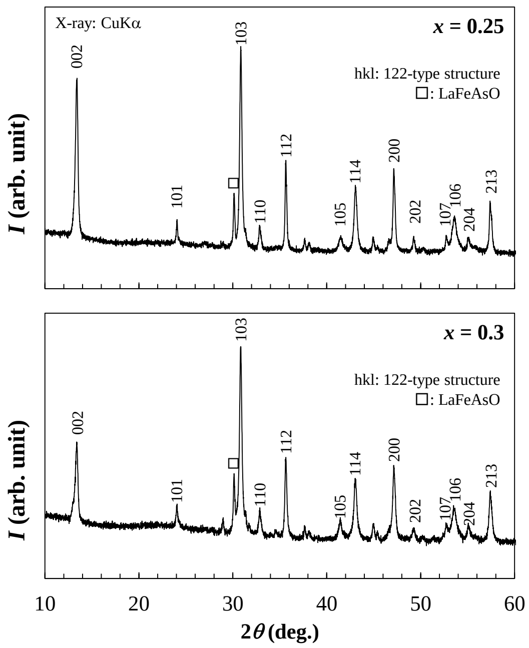

Fig. S1 powder X-ray diffraction patterns of $(La_{0.5-x}Na_xK_{0.5})Fe_2As_2$ (x = 0.25, 0.3).

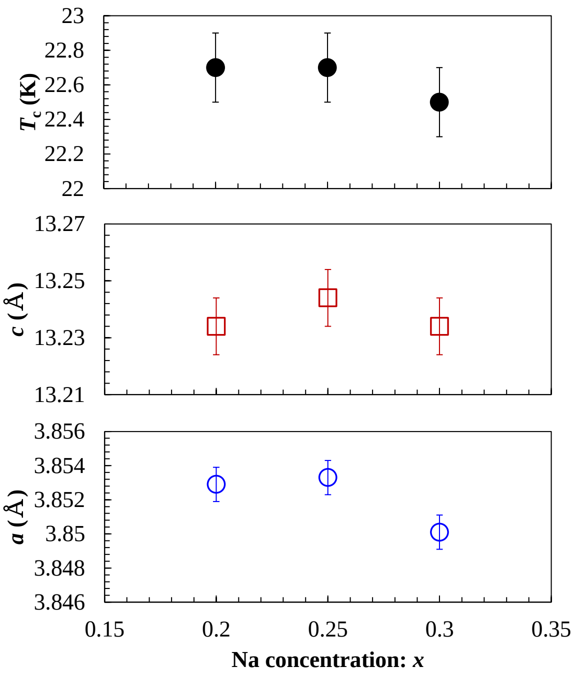

Fig. S2 Lattice constants a and c and T_c of $(La_{0.5-x}Na_xK_{0.5})Fe_2As_2$.
